# Immune Clearance of Senescent Cells to Combat Ageing and Chronic Diseases

**DOI:** 10.3390/cells9030671

**Published:** 2020-03-10

**Authors:** Ping Song, Junqing An, Ming-Hui Zou

**Affiliations:** Center for Molecular and Translational Medicine, Georgia State University, 157 Decatur Street SE, Atlanta, GA 30303, USA

**Keywords:** cellular senescence, senescence immunotherapy, ageing, chronic disease, ageing markers

## Abstract

Senescent cells are generally characterized by permanent cell cycle arrest, metabolic alteration and activation, and apoptotic resistance in multiple organs due to various stressors. Excessive accumulation of senescent cells in numerous tissues leads to multiple chronic diseases, tissue dysfunction, age-related diseases and organ ageing. Immune cells can remove senescent cells. Immunaging or impaired innate and adaptive immune responses by senescent cells result in persistent accumulation of various senescent cells. Although senolytics—drugs that selectively remove senescent cells by inducing their apoptosis—are recent hot topics and are making significant research progress, senescence immunotherapies using immune cell-mediated clearance of senescent cells are emerging and promising strategies to fight ageing and multiple chronic diseases. This short review provides an overview of the research progress to date concerning senescent cell-caused chronic diseases and tissue ageing, as well as the regulation of senescence by small-molecule drugs in clinical trials and different roles and regulation of immune cells in the elimination of senescent cells. Mounting evidence indicates that immunotherapy targeting senescent cells combats ageing and chronic diseases and subsequently extends the healthy lifespan.

## 1. Introduction

Cellular senescence is a cell state in which the cell-cycle is generally irreversibly stopped [1], with cell-cycle reentry being a plausible scenario under specific circumstances, particularly in tumor cells [2]. Cellular senescence is significantly distinct from cell quiescence, which has a reversible cell cycle arrest. Cellular senescence is also different from cell terminal differentiation accompanied by generally irreversible cell cycle arrest but without macromolecular damage [1]. There are two major types of cellular senescence: stress-induced premature cellular senescence [3] and replicative senescence due to a repeated cell cycle, which is usually mediated by telomere shortening [4]. Cellular senescence plays beneficial critical roles in numerous biological processes [5], such as tumor suppression [6,7], embryonic tissue remodeling [8] and wound healing after injury [9]. p16^Ink4a^ (encoded by the *INK4a/ARF* locus, also known as CDKN2a, hereafter referred to as p16)-induced senescence of mouse and human pancreatic beta cells also promotes insulin secretion [10]. However, excessive accumulation of senescent cells causally shortens the healthy lifespan [11] and drives organ ageing [12], age-related organ deterioration/disorders [13,14], tissue dysfunction and chronic diseases, including cardiovascular diseases (CVDs) [15,16], cancer [2], neurodegenerative diseases [17,18] and osteoarthritis [19]. Usually, sudden or acute senescence will exert beneficial functions such as anti-fibrosis [20] and wound healing [21]. Therefore, the homeostasis of cellular senescence is crucial for normal physiology. Generally, cellular senescence is caused by various intrinsic and extrinsic factors, including telomere (the repetitive sequences of DNA at the end of eukaryotic chromosomes) attrition [16], DNA damage [22,23], accumulated cytosolic DNA from the nucleus, mitochondrial and fragmented chromatin, oncogenes such as RAS and RAF [24], mitochondrial dysfunction [25] and reactive oxygen or nitrogen species (ROS or RNS) [1]. Cellular senescence accumulation usually arises from increased stresses-induced cellular senescence and reduction of senescent cell removal due to apoptosis evasion and/or immune system dysfunction. This review summarizes and discusses the latest advances concerning the different tissue-ageing and chronic diseases caused by different senescent cells, anti-ageing and chronic diseases by small-molecule drugs in clinical trials and distinct eradication of senescent cells by individual immune cells.

## 2. Cellular Senescence Causally Contributes to Ageing and Chronic Diseases

Excessive senescent cells have been demonstrated to play a causal role in driving ageing [26] and chronic diseases [18] using genetic and pharmacologic approaches. Different senescent cells with unique features have distinct functions in tissue ageing and various chronic diseases. Controlling and balancing cellular senescence may regulate the initiation and progression of both organ ageing and chronic diseases.

### 2.1. Features of Cellular Senescence

Cellular senescence presents multiple cellular and molecular features [1], which may function as suitable biomarkers or therapeutic targets. Senescent cells generally demonstrate an enlarged and flattened cell morphology [27] and expanded nucleoli [28,29], enhanced senescence-associated beta-galactosidase (SA-β-gal) activity [30], telomere shortening, elevation of the cyclin-dependent kinase inhibitor p16 [11,31] or p21 [32], macromolecular damage and metabolism dysfunction [1]. The prominent characteristic of senescent cells is the senescence-associated secretory phenotype (SASP). Senescent cells usually secrete several pro-inflammatory cytokines (such as interleukin [IL]-1, IL-6, IL-8, tumor necrosis factor [TNF]α and monocyte chemo-attractant proteins), growth factors (including platelet-derived growth factor AA [PDGF-AA] [9], vascular endothelial growth factor [VEGF] [33] and insulin-like growth factor binding proteins 4 and 7 [IGFBP4/7] [34]), chemokines and extracellular matrix-degrading proteins, including matrix metalloproteinases (MMPs) [1]. Recently, cyclic guanosine monophosphate-adenosine monophosphate (GMP-AMP) synthase (cGAS) was shown to recognize cytosolic DNA in senescent cells to produce cGAMP, which triggers the generation of SASP factors via stimulator of interferon genes (STING) and promotes paracrine senescence [35]. Another crucial feature of senescent cells is apoptotic resistance, which is, in part, attributable to the transcriptional and cap-independent translational elevation of anti-apoptotic B-cell lymphoma 2 (BCL-2) family proteins (BCL-2, BCL-X_L_ and BCL-W) [36]. Senescent cells can generate lipofuscin by aggregation of oxidized proteins with sugars and lipids [1]. Lipofuscin is an emerging and more sensitive biomarker than SA-β-gal activity for cellular senescence in vitro and in vivo. It can be visualized in lysosomes by light microscopy or a specific histochemical stain with a biotin-linked Sudan Black B (SBB) analog [37]. It is noteworthy that different senescent cells have unique features, which may lead to distinct types of ageing patterns in different individuals [38].

### 2.2. Senescent Cells Drive Ageing and Various Chronic Diseases

Senescent cells generate multiple factors that destroy tissue function, remodel tissue structure and alter the fate of neighboring cells negatively. Mounting evidence from human samples and preclinical animal models shows that the excessive presence of senescent cells is a contributing cause of ageing, which is featured by a functional decline in multiple physiological systems [39,40]. There are several hallmarks of ageing in preclinical research and clinical practice [26,38]: (1) frailty measures are widely and commonly used clinical assessments for age stages [41]. One of the two most common measurements is Fried’s frailty phenotype (known as the Cardiovascular Health Study [CHS] Index), which includes weight loss, weakness (lower grip strength [40]), slow gait speed, low physical activity (voluntary exercise for moue) and decreased exercise endurance [42]; (2) disordered circadian behavior profiles such as sleep-wake cycles [43]; (3) increased inflammaging with decreased systemic cytotoxicity [40]; (4) the epigenetic clock based on the DNA methylation profile, which is likely related to biological age other than chronological age [44]; (5) the glomerular filtration rate, which correlates inversely with age [38]; (6) other biological molecules, such as PDGF-BB and VEGF-D, which are positively related to age [38]. It is noteworthy that the pattern of the same ageing molecular marker at the population level may be different from that on the individual level. For example, the hemoglobin A1C level correlates positively with ageing at the popular level but has a negative correlation with ageing at the personal level in numerous individuals. All the results suggest that different individuals have distinct ageotypes [38]. It was reported that premature endothelium senescence reversibly triggers vascular ageing and the age-associated decrease in exercise endurance in old mice [42]. Senescent cells in patients with idiopathic pulmonary fibrosis (IPF) drive physical dysfunction [45]. T-cell senescence is profoundly associated with physical frailty [46].

Differential cellular senescence drives unique organ ageing and numerous chronic diseases, such as CVD, cancer and neurodegenerative diseases (Table 1). Senescent cardiovascular cells specifically contributes to chronic CVD, including atherosclerosis [47], abdominal aortic aneurysm (AAA) [48], thoracic aortic aneurysm (TAA) [49], artery stiffness [50] and heart failure [51]. Accumulation of senescent endothelial cells leads to cardiovascular thrombotic complications, including arterial (acute myocardial infarction, stroke) and venous (deep vein thrombosis, pulmonary embolism) thrombotic events [52]. Senescent endothelial cells potentially modulate immunosenescence [53]. Senescence of vascular smooth muscle cells (VSMCs) generally drives the vulnerability of atherosclerotic plaques resulting in myocardial infarction and stroke [54]. Additionally, macrophage senescence instigates features of plaque instability, such as elastic fiber defects and fibrous cap thinning in the brachiocephalic artery and descending aorta [47].

Senescence of the central nervous system (CNS) drives many neurodegenerative diseases [55], including Alzheimer’s disease (AD), Parkinson’s disease (PD) and other types of dementia (Table 1). Zhang et al. employed a senolytic cocktail of dasatinib plus quercetin (D + Q) to validate that senescent OLIG2- and NG2-expressing oligodendrocyte progenitor cells (OPCs) contribute to cognitive impairment in an AD mouse model [56]. Bussian et al. reported that clearance of p16^+^ senescent astrocytes and microglia using *INKATTAC* transgene restrains gliosis, super-phosphorylation of both soluble and insoluble tau resulting in neurofibrillary tangle deposition and degeneration of cortical and hippocampal neurons, thus restoring cognitive function. Pharmacological prevention with oral senolytic ABT263 (navitoclax) attenuates tau phosphorylation and aggregation [18]. These results suggest that senescent glial cells drive tau-dependent disease. Additionally, senescent astrocytes mediate dopaminergic neurodegeneration related to PD [57].

Cellular senescence traditionally impairs stem-cell function (also termed as stemness) [58]. For example, senescence factor p53 or p16 inhibits healthy cell conversion into an induced pluripotent stem cell. However, the senescence of specific cancer cells develops cancer stemness. Elegant research done by Milanovic et al. [2] demonstrated the following: (1) chemotherapeutic agent adriamycin-induced senescent BCL2 lymphomas from Eµ-Myc transgenic mice display notable upregulation of activated WNT signaling, an adult tissue stem-cell marker, compared with non-senescent cells; (2) targeting H3K9me3 or p53 to create a genetically switchable model of senescence to mimic spontaneous escape from the senescence cell cycle arrest validates that cells released from senescence re-enter the cell cycle with increased WNT-mediated clonogenic growth capability compared with non-senescent cells equally exposed to adriamycin, suggesting this tumor cell senescence is unexpectedly reversible; (3) acutely enhanced senescence in p53-regulatable models of acute lymphoblastic leukemia and acute myeloid leukemia can reprogram non-stem leukemia cells into self-renewing, leukemia-initiating stem cells, suggesting acute leukemia senescence by chemotherapy spontaneously develops aggressive and malignant tumor-initiating stemness.

Further research is needed to reveal how different senescent cells, or the same senescent cells, influence ageing and chronic diseases under differential contexts.

### 2.3. Regulation of Cellular Senescence and Consequent Tissue Ageing and Disorders

Cellular senescence can be inhibited or delayed by various factors, which may extend the healthy lifespan. Here, we discuss a few critical compounds for healthy ageing that have emerged during the past 10 years.

**Table 1:** Cellular senescence leads to chronic diseases or tissue ageing in animals and humans.

Types of Senescent Cells	Disorders and Aged Tissues	References
Adipocytes	Poor physical function, vascular dysfunction, cardiac ageing and a shorter health span and lifespan in mice	[59,60,61,62]
Astrocytes	Neuropathology related to Parkinson’s disease	[57]
Astrocytes and microglia	Cognitive decline	[18]
Beige progenitor cells	Age-related decline in beiging and thermogenesis	[63]
Beta cells	Type 1 diabetes and type 2 diabetes	[64,65]
Cardiac progenitor cells	Impaired heart regeneration	[66]
Cardiac fibroblasts	Age-related cardiac fibrosis and dysfunction	[61]
Cardiomyocytes	Cardiac ageing (fibrosis and hypertrophy) and heart failure	[67,68,69]
Cholangiocytes	Liver fibrosis	[70]
Chondrocytes	Osteoarthritis	[71]
Endothelial cells	Atherosclerosis, artery stiffness, thrombosis and heart failure with a preserved ejection fraction	[72,73,74]
Endothelial progenitor cells	Impaired neovascularization and preeclampsia	[75]
Fat progenitor cells	Lipodystrophy and fat loss in old mice	[76]
Fibroblasts	Atherosclerosis, lung fibrosis and decreased health and life span	[77,78,79]
Fibroblasts (in synovial tissue)	Rheumatoid arthritis	[80]
Glial cells	Neuropsychiatric disorders, including anxiety and depression	[81]
Hematopoietic stem cells	Immune function decline	[82]
Hepatic stellate cells	Liver fibrosis	[20,83]
Hepatocytes	Age-related hepatic steatosis	[84]
Macrophages	Atherosclerosis	[47]
Melanocytes	Human skin ageing	[85]
Muscle stem cells	Sarcopenia	[82]
Myofibroblasts	Myocardial fibrosis reduction	[86]
Neural progenitor cells (SOX2^+^)	Progressive multiple sclerosis	[87]
Oligodendrocyte progenitor cells	Cognitive deficits in Alzheimer’s disease mice	[56]
Osteocytes	Age-related osteoporosis (bone loss) in mice	[88]
T cells	Abnormal glucose homeostasis, insulin resistance, physical frailty	[46,89]
Vascular smooth muscle cells	Atherosclerosis, AAA, TAA, artery restenosis, aortic calcification, vasomotor dysfunction in aged or atherosclerotic mice	[48,49,50,54,90]

SOX2, SRY (sex-determining region Y)-box 2. For definitions of other abbreviations, please see the main text.

The availability of oxidized nicotinamide adenine dinucleotide (NAD^+^) decreases with age and under certain disease conditions [91]. NAD^+^ is usually generated by the kynurenine pathway of tryptophan catabolism [92] and salvage pathway [93]. NAD^+^ precursor nicotinamide riboside (NR) prevents muscle stem cell senescence by improving mitochondrial function [94]. Treatment with NR rejuvenates muscle stem cells in old (aged 22–24 months) mice by inducing the mitochondrial unfolded protein response and synthesis of prohibitin proteins. Moreover, NR delays the senescence of neural stem cells and melanocyte stem cells and enhances the mouse lifespan [94]. Oral administration of nicotinamide mononucleotide (NMN), an essential NAD^+^ precursor, to regular chow diet-fed wild-type C57BL/6N mice for 12 months remarkably and effectively mitigates age-related pathological alterations in mice without any noticeable side effects. For example, NMN suppresses age-associated body weight gain, promotes physical activity and improves insulin sensitivity, plasma lipid profile, eye function, tear production and bone mineral density [91]. NMN treatment also improves blood flow and increases endurance in old (aged ~20–22 months) mice by promoting sirtuin deacetylase SIRT1-mediated induction of capillary density, an effect synergized by exercise [42]. Interestingly, a recent clinical trial (NCT03151239) for NMN safety in humans reported that single oral administration of NMN shows no significant clinical symptoms or alterations in the heart rate, blood pressure and body temperature, suggesting the single oral supplementation of NMN is safe and effectively metabolized in healthy men [95]. However, the potential therapeutic strategy of NMN for anti-ageing and chronic diseases needs to be further explored and characterized.

It was reported that mitochondria-targeted gasotransmitter hydrogen sulfide (H_2_S) delays endothelium senescence [96]. Recently, substantial evidence indicates that H_2_S exerts a potential evolutionarily conserved function of anti-vascular ageing [42]. H_2_S plays this role via the regulation of endothelial NAD^+^ levels [42] or post-translational modification of reactive cysteine residues by protein persulfidation (S-sulfhydration) [97]. Thus, the H_2_S generator sodium hydrosulfide could battle vascular ageing and chronic diseases.

Rapamycin decelerates cellular senescence in vitro and in vivo via mechanistic target of rapamycin (mTOR). Recently, rapamycin was reported to exert in vivo neuroprotective and anti-ageing effects via the activation of lysosomal mucolipin TRP channels, independent of mTOR [98]. Moreover, rapamycin, the US Food and Drug Administration-approved mTOR inhibitor, has been shown to extend the median and maximal lifespans of both male and female genetically heterogeneous mice [99]. Twenty-four-month-old female C57BL/6J mice treated with rapamycin for 3 months present improved late-life vascular contractile function and antihypertrophic signaling in the aged heart with remission in age-associated inflammation. Rapamycin treatment also results in beneficial behavioral, skeletal and motor changes in old mice [100]. A phase 2a clinical study with a low-dose combination of a catalytic (BEZ235) plus an allosteric (RAD001) mTOR inhibitor that selectively inhibits mTOR downstream target of rapamycin complex 1 (TORC1) for 6 weeks demonstrates that mTOR inhibitor therapy is safe, enhances immune function, decreases the incidence of upper respiratory infections and improves the response to influenza vaccination in seniors [101]. The clinical trial NCT03103893 indicates that topical rapamycin treatment for 6~8 months decreases p16 expression associated with reduced cellular senescence of human skin and improves the clinical signs of ageing with increased collagen VII expression in the skin [102].

Metformin, a widely prescribed first-line oral drug to treat type 2 diabetes, inhibits cellular senescence in vitro and in animal models via multiple molecular and cellular mechanisms [103,104,105]. For example, metformin inhibits oncogene-induced SASP by blocking nuclear factor-κB activation in human diploid fibroblasts [106]. Interestingly, metformin has been reported to extend the healthspan in Caenorhabditis elegans via the liver kinase B1/5′ AMP-activated protein kinase pathway [107] or changing microbial folate and methionine metabolism [108]. Recently, Pryor et al. reported that gut microbes integrate nutrition to regulate metformin effects on host longevity through the transcriptional regulator cAMP response protein (CRP)-mediated phosphotransferase signaling pathway. They predicted the bacterial production of agmatine (a product of arginine metabolism), a mediator of metformin effects on host fatty acid metabolism and lifespan extension [109]. The first clinical trial (phase 4) of the metformin effect on the biology of human ageing was launched as “The Metformin in Longevity Study (MILES)” in 2014. The results indicate that metformin modulates metabolic and nonmetabolic gene expression in skeletal muscle and subcutaneous adipose tissues of older persons [110]. The phase 2 clinical trial (NCT02570672) “Metformin for Preventing Frailty in High-risk Older Adults,” which considered frailty as a vital endpoint, was undertaken since 2015 [111]. Another phase 4 clinical study (NCT02915198) investigating the outcome of metformin in patients with pre-diabetes and established atherosclerotic cardiovascular disease started in February 2019. The recent multicenter trial “Targeting Ageing with Metformin (TAME)” focusing on targeting ageing and chronic conditions may be supported by NIH in the future [112]. Given the uncertain side effects (vitamin B12 deficiency, lactic acidosis and gastrointestinal side effects) of metformin presenting in some individuals [104], personal metformin therapy for anti-ageing may demand more in-depth study.

In addition, β-hydroxybutyrate (β-HB) may mediate the effect of a ketogenic diet [113,114], intermittent fasting [115] or exercise [116] on healthspan extension or neuroregeneration because of its anti-inflammation [117], inhibition of vascular cell senescence [118] or immune activation by the formation and maintenance of CD8^+^ memory T cells [119]. It would be interesting to test the action of β-HB on healthy ageing and chronic diseases in preclinical and clinical research. Interestingly, recent clinical trials with senolytics D + Q demonstrate promising results. The first-in-human open-label pilot study indicates that D + Q directly eliminates senescent cells in human adipose tissue and skin [120] and significantly improves the physical function of participants with idiopathic pulmonary fibrosis [45]. These results suggest that the strategy of combining treatments targeting different senescent cell populations with distinct phenotypes would be valid for anti-ageing and chronic diseases.

## 3. Immunosurveillance of Senescent Cells

Generally, senescent cells can be removed by apoptosis and the immune system [121,122]. Because apoptosis evasion features most senescent cells, the immune system, including adaptive and innate immune cells, plays a critical role in the eradication of senescent cells at a young stage or under physiological conditions. Although senescent cells can be induced to undergo apoptosis by senolytics [45,60,78,81,120], these apoptotic cells must be finally cleared by the immune system. Interaction between senescent and immune cells affects immune system function. Senescent cells recruit and make immune cells senescent and dysfunctional via SASP, leading to persistent and excessive accumulation of senescent cells [122]. However, the precise mechanism underlying senescent cell accumulation within tissues is still debatable. It is unknown whether this is due to senescent cell increase exceeding the immune system’s ability to clear them or immune cell dysfunction.

### 3.1. Maintenance of Immune System Function to Keep Healthy Longevity

A study investigating human ageing at the individual level by frequent sampling and prolonged deep molecular profiling indicates that immune pathways are one of the significant pathways that alter with age [38]. Males generally have a shorter average lifespan than females partly due to fewer B cells and weaker B-cell-mediated humoral immunity inhibited by the CCL21-GPR174-Gαi pathway [123]. With ageing, human immune cells become senescent (known as immunosenescence). Many functions of the immune system progressively decline with age (younger than 100 years). For example, the number of inhibitory receptor natural killer group 2A (NKG2A)-positive CD8^+^ T cells in the blood of healthy volunteers dramatically increases with age. These highly differentiated CD8^+^ T cells can be inhibited by human leukocyte antigen (HLA)-E generated by senescent fibroblasts and the endothelium [124]. It was reported that aged mice [125] and humans [125,126] have an increased proportion and suppressive activity of FOXP3^+^ regulatory T (T_regs_) cells, which suppress T effector cell function. Interestingly, a recent study using single-cell RNA analysis for circulating immune cells demonstrated that supercentenarians older than 110 years with healthy ageing have an increased number of CD4^+^ cytotoxic T lymphocytes (~25.3% of total T cells) compared with only 2.8% of all T cells in young controls (~50–80 year olds), while both the supercentenarians and control groups have almost the same number of T cells. These CD4^+^ cytotoxic T cells in supercentenarians are produced by clonal expansion and have an identical transcriptome as cytotoxic CD8^+^ T cells. However, the supercentenarians have a dramatic reduction of the B-cell number compared with controls [127]. These immune signatures of supercentenarians well explain that the increased healthy longevity is due to immunosurveillance of some conditions such as infections [128] and tumor development [129].

### 3.2. Immunotherapy to Eliminate Senescent Cells

**Figure 1:**
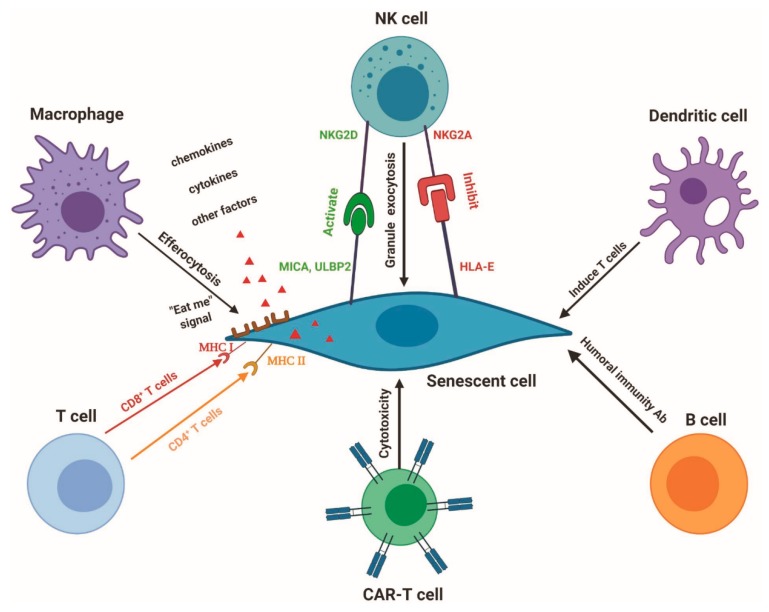
Senescent cell elimination by the immune system. Different senescent cells generate distinct ligands, including MICA/B, ULBP2, MHC I, MHC II and HLA-E. Individual immune cells, including macrophages, NK cells, T cells, CAR-T cells and dendritic cells, can precisely recognize the senescent cells via these ligands and targeted eradicate senescent cells. B cells may attack senescent cells through a humoral immunity antibody.

Mounting evidence demonstrates that immune surveillance of senescent cells is executed by different immune cells such as macrophages, natural killer (NK) cells and cytotoxic T cells in cancer [5,130] and chronic liver cirrhosis [20]. Different senescent cells generate distinct ligands that attract individual immune cells for immunosurveillance (Figure 1). For example, senescence-activated hepatic stellate cells upregulate cell-surface MICA and ULBP2, ligands of activating receptor NKG2D on NK cells [20]. Presently, senescence immunotherapy is an emerging research arena [20,131,132,133]. Senescence immunotherapy strategy is also a promising alternative to senolytics to remove senescent cells in the prevention and cure of ageing and chronic diseases (Table 2). Different immune cells have a distinct capability to identify and eliminate the unique senescent cells. Here, we discuss the roles and regulation of vital immune cells in combating chronic diseases and ageing.

#### 3.2.1. Macrophages

Specific populations of innate immune effector macrophages take up apoptotic cells in particular tissues, known as efferocytosis, which prevents apoptotic cells from being necrotic or pro-inflammatory [138]. The impaired ability of macrophage efferocytosis would enhance atherogenesis [139,140,141] and atherosclerotic plaque instability [142,143,144]. For example, interferon regulatory factor 5 (IRF5) transcription factor enhances vulnerable plaque formation in hyperlipidemic apolipoprotein E-deficient (ApoE^−/−^) mice through the induction of pro-inflammatory CD11c^+^ macrophages within atherosclerotic lesions and increasing the expansion of the necrotic core by inhibiting macrophage efferocytosis [142].

Although senescent cells can be induced to undergo apoptosis by senolytics and further removed by macrophages, macrophages also directly engulf senescent cells in cancer (Figure 1). For example, p53 reconstitution induces liver tumor cell senescence with increased p16 and SA β-gal activity but not apoptosis, in mice in vivo. These senescent tumor cells recruit innate immune cells, such as macrophages, leading to the eradication of senescent tumor cells and subsequent tumor regression [145]. Kang et al. demonstrated that CD4^+^ T cells require monocytes/macrophages but not NK cells, to remove pre-malignant senescent hepatocytes, subsequently blocking liver tumor development [130]. F4/80^+^ macrophages are also key players in the removal of senescent uterine cells after parturition to keep postpartum uterine functionality in wild-type mice and maintain the success rate of a second pregnancy in a preterm birth mouse model [135]. Whether macrophages remove senescent cells in aged or diseased systems remains to be elucidated.

Both macrophage and apoptotic or senescent cells control macrophage efferocytosis ability. Macrophages in the peritoneum, pleural cavity and lung alveoli constantly and efficiently engulf apoptotic cells at a steady state. The local tissue microenvironment programmes these macrophages with restricted responses to low doses of nucleic acid within apoptotic cells and lacking expression of toll-like receptor 9 (TLR9). Macrophage transcription factors Kruppel-like factors 2 (KLF2) and 4 (KLF4) are crucial controllers inducing gene expression necessary to silently eradicate apoptotic cells [138]. Recently, Yang et al. reported that the C-type lectin receptor LSECtin (Clec4g) on colon macrophages is required for macrophage engulfment and clearance of apoptotic cells, contributing to intestinal repair in dextran sulphate sodium-induced colitis [146]. Interestingly, activated Treg cells secrete IL-13 to stimulate IL-10 production in macrophages via binding to the IL-13 receptor. The elevated IL-10 signaling upregulates macrophage STAT3-mediated VAV1 (a guanine nucleotide exchange factor), which activates GTPase Rac1 to enhance apoptotic cell engulfment by macrophages [139]. It is noteworthy that the sustainable clearance of multiple apoptotic cells by macrophages requires dynamin-related protein 1 (DRP1)-mediated macrophage mitochondrial fission, allowing calcium release from mitochondria into the cytoplasm, triggered by the initial uptake of apoptotic cells [147]. DRP1-deficient macrophages present impaired efferocytosis in vivo and subsequently increased plaque necrosis in advanced atherosclerotic lesions of Western diet-fed LDLR knockout mice [147]. However, apoptotic cell fate also regulates macrophage efferocytosis ability. For example, apoptotic or senescent cells have increased expression of cell-surface protein CD47, one of the “don’t eat me” signals, which impairs efferocytosis via binding to the inhibitory receptor signal regulatory protein alpha (SIRPα) on the macrophage. Antibodies blocking CD47 reactivate efferocytosis of diseased vascular tissue without altering cellular apoptosis, as well as alleviate atherosclerosis in both the aortic sinus and en face of the aorta in multiple mouse models [148]. Additionally, cyclin-dependent kinase inhibitor 2B (CDKN2B)-deficient apoptotic cells show decreased expression of calreticulin [149], which is a principal ligand required for engulfment activation via binding and activating LDL-receptor-related protein (LRP) on macrophages [150]. Thus, apoptotic cells without CDKN2B impair macrophages efferocytosis and result in the development of advanced atherosclerotic plaques with large necrotic cores. Moreover, supplementation with exogenous calreticulin restores the clearance of CDKN2B-deficient apoptotic cells by macrophages [149]. Therefore, it is crucial to understand the molecular mechanisms regulating macrophage phagocytic ability and apoptotic/senescent cell clearance in the progression and therapy of chronic diseases and organ ageing.

#### 3.2.2. Roles and Regulation of NK Cells in Senescent Cell Removal

NK cells are involved in the elimination of senescent cells through interaction between the activating NKG2D receptor and its ligands expressed on senescent cells. For example, the YT human NK cell line preferentially destroys senescent IMR-90 fibroblasts [20]. This selectivity is due to selective upregulation of NKG2D ligands MICA and ULBP2 in senescent IMR-90 cells but not growing or quiescent cells [151]. Importantly, perforin- and granzyme-containing granule exocytosis but not death-receptor-mediated apoptosis, is required for NK cell-mediated killing of senescent cells [40]. Thus, mice with defects in granule exocytosis accumulate senescent stellate cells and display stronger liver fibrosis in response to a fibrogenic agent [131].

The ability of NK cells to kill senescent cells is controlled by multiple factors such as ligands from senescent cells. NKG2D receptor deletion instigates the accumulation of senescent stellate cells, resulting in increased liver fibrosis in mice in vivo [151]. Furthermore, NK cell activation by polyinosinic-polycytidylic acid [152] decreases the senescent cell number in the liver in vivo, leading to reduced liver fibrosis [20]. Chemotherapeutic drugs, including doxorubicin, melphalan and bortezomib, upregulate both the DNAX accessory molecule-1 (DNAM-1; CD226) ligand PVR (poliovirus receptor; CD155) and NKG2D ligands (MICA and MICB) on multiple myeloma cells presenting a senescent phenotype. These ligands enhance NK cell susceptibility [137]. Pereira et al. recently reported that senescent human primary dermal fibroblasts and endothelial cells express higher levels of the atypical major histocompatibility complex (MHC) Ib molecule HLA-E via the p38 signaling pathway than non-senescent cells. HLA-E expression is also increased in the senescent fibroblasts of human skin during ageing. HLA-E then inhibits immune cytotoxicity targeting senescent cells by interacting with the inhibitory receptor NKG2A expressed on NK cells [124]. However, p53-expressing senescent liver tumor cells recruit NK cells by inducing the chemokine CCL2 but not CCL3, CCL4 or CCL5, in senescent tumor cells without affecting the ligand expression of RAE-1 proteins in the tumor cells [153]. Collectively, agents blocking the interaction between HLA-E and NKG2A [124], the humanized anti-NKG2A monoclonal antibody monalizumab [154] and NKG2A protein expression blockers [155] would be promising strategies for the immune clearance of senescent cells and subsequent anti-ageing and chronic diseases. Additionally, chimeric antigen receptor (CAR)-NK cells may be a valuable tool for senescence immune surveillance.

#### 3.2.3. CAR-T Cells

T cells play crucial roles in immune surveillance and healthy longevity. CD4^+^ T cells generally regulate the immune response via multiple cytokines. CD4^+^ cytotoxic T cells can directly kill senescent tumor cells by recognizing MHC II molecules, which are usually absent in healthy cells but present in a subset of tumor or senescent cells [130]. CD8^+^ cytotoxic T cells directly eradicate target cells using cytotoxic molecules via recognizing MHC I molecules within nearly all cells [127]. However, the selectivity and efficiency of cytotoxic T cells decline with age. Reinstructing cytotoxic T cells to identify specific antigens on cancer cells using either a modified T-cell receptor or a chimeric antigen receptor (CAR) has been successfully used for specific cancer therapies [156]. It was reported that high expression of fibroblast activation protein (FAP) in active cardiac fibroblasts but not cardiomyocytes, drives abundant cardiac fibrosis and consequent myocardial disease [134]. Recently, the adoptive constitution of engineered specific CAR-CD8^+^ T cells selectively targeting FAP notably alleviates cardiac fibrosis and reverses both systolic and diastolic cardiac function in angiotensin II and phenylephrine-treated mice [134]. Importantly, c-Jun overexpression protects CAR-T cells from dysfunction by inducing T-cell exhaustion resistance [157]. Because senescent cells generate specific cell-surface antigen, such as band 3 [158], it is very promising to develop specific CAR-T cells for the selective depletion of senescent cells. Notably, ligands or antigens to activate the receptors NKG2D and DNAM-1 or ligands HLA-E for the inhibitory receptor NKG2A, have demonstrated expression in senescent cells. Therefore, it may be possible to target senescent cells by engineering T cells expressing NKG2D-CAR (NKG2D-CAR-T cells) that recognize NKG2D ligands on the surface of senescent cells based on cancer research [159].

#### 3.2.4. Dendritic Cells

Dendritic cells (DCs), a professional phagocytic cell type, can also identify and eradicate apoptotic cells [160]. Notably, CD24^+^ DCs generally exert their removal function via T-cell regulation. For example, CD103^+^ DCs selectively carry apoptotic intestinal epithelial cells to mesenteric lymph nodes, which function as critical determinants to induce tolerogenic CD4^+^ regulatory T-cell differentiation in mice [161]. CD11b^+^ DCs with dysfunctional autophagy due to Atg16l1 deficiency expand aortic CD4^+^ Treg cells and inhibit atherogenesis in Ldlr^−/−^ mice [162]. Indoleamine 2,3-dioxygenase 1 (IDO1)-expressing and chemokine (C-C motif) receptor 9 (CCR9)-positive plasmacytoid dendritic cells (pDCs) in the aorta locally induce aortic Treg cells, which produce IL-10 and subsequently prevent atherogenesis in mice [163]. Theoretically, senescent cells may produce specific cell-surface antigens and then DCs process and express these antigens on the cell surface that are then identified by T cells. However, it remains extremely unknown whether and how DCs eliminate apoptotic or senescent cells to prevent the development of chronic diseases, including atherosclerosis.

## 4. Conclusions and Future Perspectives

Excessive and persistent accumulation of different senescent cells drives unique ageing and chronic disease development. Because senescent cells are also beneficial in the short term, the homeostasis of senescent cells is critical for healthy ageing. Several small-molecule drugs and senolytics have been entered into clinical trials to combat cellular senescence-associated ageing and chronic diseases. Although immune elimination of distinct senescent cells is an emerging and promising strategy for anti-ageing and the therapy of different chronic diseases, such immunotherapy is not cost-free and does have side effects. To translate this novel strategy into the clinic, we plan to carry out the following investigations: 1) discovering suitable molecular biomarkers or pathways for personal ageing in preclinical and clinical research; 2) identifying unique antigens or ligands of senescent cells for immunosurveillance; 3) including both genders for research because sex differences are present for ageing and chronic diseases [123,164].

## Figures and Tables

**Table 2:** Elimination of senescent cells by immune cells.

Immune Cells	Eliminated Senescent Cells	Outcomes In Vivo	References
CAR-T cells	Fibroblasts	Fibrosis reduction in mouse heart	[134]
CD4^+^ T cells	Murine hepatocytes	Suppression of mouse liver cancer	[130]
CD8^+^ T cells	Fibroblasts	N/A	[124]
Macrophages	Uterine senescent cells	Maintain postpartum uterine function in mouse	[135]
NK cells	Hepatic stellate cells	Liver fibrosis resolution in mouse	[20]
NK cells (in uterine)	Decidual cells (endometrial stromal cells)	Endometrial rejuvenation and remodeling at human embryo implantation	[136]
NK cells	Myeloma cells	Tumor suppression in mouse	[137]
NK cells	Fibroblasts	N/A	[124]

N/A, not available.
